# Case of Azoturia in a Dog1Read before the May meeting of the Veterinary Medical Association of New York County.

**Published:** 1899-06

**Authors:** Robert Dickson


### REPORTS OF CASES.

### CASE OF AZOTURIA IN A DOG.[1]

By Robert Dickson, D.V.S.

The two following cases which came to me for treatment during the past few months being new to me, I thought it might be interesting to the members of the Society to hear the symptoms shown, treatment prescribed, and the termination of each, as I have never heard or read of any case in canine practice showing the symptoms shown in these cases.

The May number of last year's *Review* quotes a paper read before the Wisconsin Association of Veterinary Graduates of Milwaukee on "Azoturia in the Dog," by Dr. Leech, and as there is such a difference between his experience and mine I hope there will be a chance for us all to learn something.

He states "it is characterized by tonic and clonic spasm of the pelvic and lumbar muscles; also those of the pectoral region." He also states that the primary cause is dietetic, and that dogs are very liable to it, as they are very much abused for want of exercise in the proper manner; that owners also err in the way of feeding, not paying enough attention to the condition and vocation of the animals, and in that way leaving the dog more subject to the disease. Then he goes on and gives a brief description of the symptoms he found in two cases, one dog being attacked in the right pelvic limb, the other losing the power of both pelvic limbs, high-colored urine, with nitrogenous and albumin slightly present. He states they yield very easily to treatment, quickly recovering, with very little danger of their terminating fatally. How near he is right or wrong it is for you, gentlemen, to determine, or whether he has had a true case of azoturia.

The following is my experience: On February 15th I was called to see a black spaniel, nine years old, weighing twenty-one pounds, very fat and well cared for. He had an attack of nephritis last fall, and one attack a year ago last fall. He was well fed from the kitchen, getting a taste of everything he would eat. It was a standing rule for him to be exercised twice a day, morning and night. As he was taken from the house this day, the house being situated at Seventy-fourth Street and Avenue Park, he played, ran,

[1] Read before the May meeting of the Veterinary Medical Association of New York County.

jumped, and appeared to feel as good as usual until he reached Fifth Avenue and Seventy-fifth Street, when he was suddenly taken with a faulty gait, reeling and staggering, and finally falling down, becoming unable to walk. He was carried home and I was sent for. On arriving at the house I found him lying on his left side. He appeared bright and showed no symptoms of any pain, but could not stand up. Thinking it a case of acute paralysis from some old kidney trouble, I prescribed accordingly. Gave cathartic, but, to my surprise, the next day found he had died at 9 o'clock, after twenty-three hours of sickness.

My second case was a black-and-tan, common-bred dog, two years and six months old, weighing forty pounds. He had a short, thick body, and was very fat. He was kept in the house and store as a night watch-dog, but was never tied up; was allowed to run in the streets a few minutes three times a day on dry days; on wet days was put in a small, covered yard. He was very choice and dainty about his eating; would not eat anything but choice beef and chicken, and was fed three times a day as regular as his owner ate himself.

On March 10th his appetite became capricious and diminished, increased thirst, but diminished urination, and complete constipation. Prescribed a cathartic and Sanmetto, giving two drachms every three hours. On March 11th he was very unsteady upon his feet, and when compelled to walk would reel from side to side and show symptoms of acute vertigo. When lying he appeared stupid or semi-conscious; no feces or urine voided during the day; a partial paralysis now appeared in the posterior quarters, with a twitching of the superficial voluntary muscles. Countenance became anxious, patient moaning almost constantly. The catheter was introduced during the evening. Urine evacuated was of a dark, brownish-black color. The lumbar muscles now became hard, rigid, and prominent, and the appetite impaired. Temperature 103° F., pulse 98, respiration 25.

On March 12th complete paralysis of the back loins and posterior extremities, the muscles being hard, prominent, and painful upon manipulation; the bowels operating freely, due to the cathartic administered; urine retained; catheter introduced and the urine found to be slightly changed in color. During the day the patient showed colicky pains at frequent intervals, the paroxysm lasting for four or five minutes. Temperature 103.2° F., pulse 96, respiration 28.

On March 13th, no apparent change in condition; appetite failing; bowels operating; sat up and passed urine without aid, which was of light color. Temperature 102° F., pulse 92, respiration 20. Symptoms about the same as on the previous day, the colicky pain having subsided. On March 14th he died in the morning before I arrived, struggling in his agony.

In conclusion, I would like the opinion of the members present. In my diagnosis did I have azoturia—that mysterious disease of the horse—or was it acute or chronic nephritis, meningitis, spinal meningitis, cystitis, or some as yet unidentified disease?

